# Supplementary material for: Alpha interbrain synchrony during mediated interpersonal touch
Source: PLoS One. 2024 May 17;19(5):e0300128. doi: 10.1371/journal.pone.0300128 (PMC11101020; doi:10.1371/journal.pone.0300128)

S1 Appendix. Alpha interbrain synchrony

Fig 8. Alpha interbrain synchrony. Red solid and blue dashed lines represent PLV index increased and decreased respectively during TOI compared to baseline, Wilcoxon signed-rank test, uncorrected $p < 0.01$.

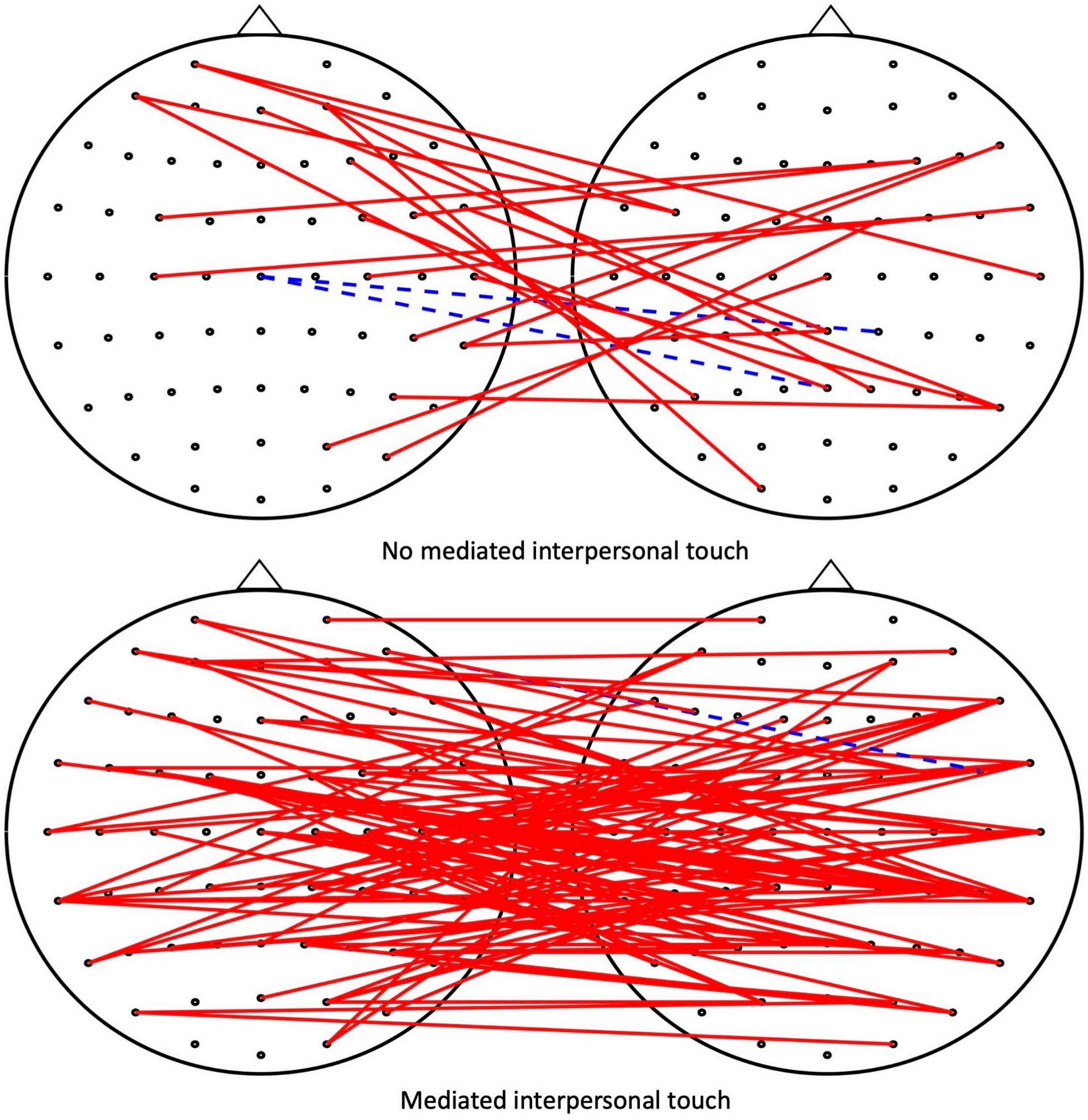

Supplement: S1 Appendix — (PDF) [file pone.0300128.s001.pdf]
