# Supplementary material for: Alpha interbrain synchrony during mediated interpersonal touch
Source: PLoS One. 2024 May 17;19(5):e0300128. doi: 10.1371/journal.pone.0300128 (PMC11101020; doi:10.1371/journal.pone.0300128)
Supplement: Attachment — Submitted filename: Response_Hyperscanning PLOS ONE.pdf [file pone.0300128.s002.pdf]

**Thank you for your kind comments about our study for the purpose of improvements. We carefully reviewed and revised this manuscript based on your comments.**

Journal Requirements:

When submitting your revision, we need you to address these additional requirements.

1. Please ensure that your manuscript meets PLOS ONE's style requirements, including those for file naming. The PLOS ONE style templates can be found at

https://journals.plos.org/plosone/s/file?id=wjVg/PLOSOne_formatting_sample_main_body.pdf and

https://journals.plos.org/plosone/s/file?id=ba62/PLOSOne_formatting_sample_title_authors_affiliations.pdf

Thank you for the comments. We have revised the manuscript to reflect the PLOS ONE’s style requirements.

2. Please amend your current ethics statement to address the following concerns:

a) Did participants provide their written or verbal informed consent to participate in this study?

b) If consent was verbal, please explain i) why written consent was not obtained, ii) how you documented participant consent, and iii) whether the ethics committees/IRB approved this consent procedure

Thank you for the comment. Participants provided their written consent by signing a consent form presented to them before the experiment. All the human subject experimental protocols are duly approved by the IRB ethics committee at NYUAD including the written consent form contents.

To make it more visible we have modified the “Participants” section under the “Material and Methods” section

3. Thank you for stating the following in the Acknowledgments Section of your manuscript:

"This work is supported by the NYUAD Center for Artificial Intelligence and Robotics (CAIR), funded by Tamkeen under the NYUAD Research Institute Award,"

We note that you have provided additional information within the Acknowledgements Section that is not currently declared in your Funding Statement. Please note that funding information should not appear in the Acknowledgments section or other areas of your manuscript. We will only publish funding information present in the Funding Statement section of the online submission form.

Please remove any funding-related text from the manuscript and let us know how you would like to update your Funding Statement. Currently, your Funding Statement reads as follows:

"YES. M.E. received the funding. This work is supported by the NYUAD Center for Artificial Intelligence and Robotics (CAIR), funded by Tamkeen under the NYUAD Research Institute Award, CG010. URL for funding agency: https://www.tamkeenuae.com/"

Please include your amended statements within your cover letter; we will change the online submission form on your behalf.

Thanks for the comment. We removed the funding statement from the acknowledgment section and provided the exact wording within the cover letter for inclusion in the Funding Statement.

4. Please include your full ethics statement in the 'Methods' section of your manuscript file. In your statement, please include the full name of the IRB or ethics committee who approved or waived your study, as well as whether or not you obtained informed written or verbal consent. If consent was waived for your study, please include this information in your statement as well.

Thank you for your comment. All the human subject experimental protocols are duly approved by the NYUAD Institutional Review Board (NYUAD IRB) ethics committee, including the written consent form contents. Participants provided their written consent by signing a consent form presented to them before the experiment. We modified the "Participants" section under the "Material and Methods" section to clarify these points.

5. Please include captions for your Supporting Information files at the end of your manuscript, and update any in-text citations to match accordingly. Please see our Supporting Information guidelines for more information: http://journals.plos.org/plosone/s/supporting-information.

[Note: HTML markup is below. Please do not edit.]

Thanks for the comment. Done.

Reviewers' comments:

Reviewer's Responses to Questions

**Comments to the Author**

1. Is the manuscript technically sound, and do the data support the conclusions?

The manuscript must describe a technically sound piece of scientific research with data that supports the conclusions. Experiments must have been conducted rigorously, with appropriate controls, replication, and sample sizes. The conclusions must be drawn appropriately based on the data presented.

Reviewer #1: Partly

Reviewer #2: No

---

2. Has the statistical analysis been performed appropriately and rigorously?

Reviewer #1: Yes

Reviewer #2: No

We have updated the statistical analysis to incorporate corrections for multiple comparisons. We hope that these corrections are in line with the reviewers' expectations and the PLOS ONE standard.

---

3. Have the authors made all data underlying the findings in their manuscript fully available?

The PLOS Data policy requires authors to make all data underlying the findings described in their manuscript fully available without restriction, with rare exception (please refer to the Data Availability Statement in the manuscript PDF file). The data should be provided as part of the manuscript or its supporting information, or deposited to a public repository. For example, in addition to summary statistics, the data points behind means, medians and variance measures should be available. If there are restrictions on publicly sharing data—e.g. participant privacy or use of data from a third party—those must be specified.

Reviewer #1: Yes

Reviewer #2: No

We have shared the data through the OSF. The link is mentioned in the Data Availability section.

---

4. Is the manuscript presented in an intelligible fashion and written in standard English?

PLOS ONE does not copyedit accepted manuscripts, so the language in submitted articles must be clear, correct, and unambiguous. Any typographical or grammatical errors should be corrected at revision, so please note any specific errors here.

Reviewer #1: Yes

Reviewer #2: Yes

---

5. Review Comments to the Author

Please use the space provided to explain your answers to the questions above. You may also include additional comments for the author, including concerns about dual publication, research ethics, or publication ethics. (Please upload your review as an attachment if it exceeds 20,000 characters)

**Reviewer #1:**

In this study, the researchers examined the neural synchrony during mediated touch. Paris of strangers each pressed and received pressure from a sleeve and their neural activity was measured. Researchers found increased synchrony in the alpha band during squeezes. This study is well designed and well analyzed. I think the results can contribute to the field of mediated touch and its neural underpinnings. However, as the manuscript is written now it is unfit for publication. The authors need to elaborate and pick better references for their claims in the introduction section. They need to better define their hypothesis and add about their task and why they chose to design it. The whole discussion section is written as a technical description of the task and the results with almost no mention of the meaning of the results, their significance, and their contribution to the field. It is almost written as a review of a new technological device and not an academic paper.

Abstract:

1. Please add a sentence or two about interpreting your findings and their significance.

Thanks for the comment. We added a sentence to indicate the interpretation of the findings (the effect of social coordination).

2. Add abbreviation (PLV) after introducing the term for the first time.

Thanks for the comment. We added the abbreviation in the abstract.

Introduction:

1. There is a mismatch between some of the papers the authors cited to the claim they make in the introduction:

a. Citing the Eid et al., 2015 when talking about intimacy of touch is not right here since this paper is about haptic touch – it is more appropriate later when talking about haptic touch.

The citation is a literature review covering several studies supporting the statement. To make it explicit, we cite another study that explicitly supports the statement.

b. Citing the Dunbar 1991 when talking about handshaking in social interactions is not right since this paper is about back grooming in primates and how it is social and not just hygienic – there are more suitable papers on human touch to cite here.

We did not mean to single out handshaking but rather give an example of social interaction. To avoid the confusion, we deleted the word handshake.

c. Erl et al., 2015 talks about mediated social touch and does not talk about "kissing hugging handholding or cuddling" in any way.

Thanks for the catch. We intended to reference the affective haptics literature review here. We corrected the reference.

2. "touch can convey vitality and immediacy more powerfully than language" – can the authors elaborate on that? Especially since it may be connected to their topic of the study.

We added the following examples to clarify the statement "such as a strong handshake or a nudge for attention" in the Introduction section.

3. "touch is commonly utilized to enhance the meaning of other forms of verbal and non-verbal communications" – please elaborate on that as well here.

Thanks for the comment. What is meant here is that touch amplifies the intensity of other non-verbal responses (such as emotional response from our face or voice). We added an explanation to this statement.

4. Please explain the Midas touch effect for the readers who are not familiar with the literature on that

Thanks for the comment. We provide an example to explain the Midas touch effect.

5. Please explain the “negative connotations” of touch you mentioned.

Thanks for the comment. We mean several wellness issues including physiological, cognitive, social, communication, and emotional well-being. We added these clarifications to the sentence.

6. Please cite Goldstein et al., 2018 along with your citation of Kaus et al., 2020 – about touch and hyperscanning.
Goldstein, P., Weissman-Fogel, I., Dumas, G., & Shamay-Tsoory, S. G. (2018). Brain-to-brain coupling during handholding is associated with pain reduction. Proceedings of the national academy of sciences, 115(11), E2528-E2537.

Thanks for providing the highly relevant reference. We added it to the same paragraph.

7. Explain what is “phase locking value” and why is it good to use it here to measure your variables.

Thanks for the comment. We provided further explanation to the PLV concept and how it works, along with a citation for the article that introduced it.

8. Be more specific about your hypothesis – why would MIT increase interbrain synchrony? What are the measures that will be larger? Compared to what will it be larger? Please justify your study and its meaning and significance more in the introduction. At the moment it is a bit lacking although I do think this topic is of interests, its just needs to be expressed better.

Thank you for the valuable comment. We modified the last paragraph of the introduction section to clarify the hypothesis and significance of the study. We also provide a summary of the findings.

Methods:
1. Please provide mean and s.d of age of the participants.

We did not collect the exact age as our IRB policy does not permit exact age information, so we collected age group data. Undergraduate students aged 18-24 were enrolled and also listed under the “Participants” section.

2. What is the rationale for having participants who are unfamiliar with one another?

Thank you for the comment. The reason for this is to minimize any potential bias while performing the task. We anticipate a certain level of coordination/synchronization between the participants’ brains during the haptic task. If two individuals are familiar with each other, it may influence the execution of the task, as one participant may unconsciously apply less or more pressure on the sleeve, knowing that the other person is their friend or acquaintance.

To make it more visible we have modified the “Participants” section under the “Materials and Methods” section.

3. Were the pairs mixed gender or same gender? Or both?

There was no restriction on the pairing of the gender. Both genders could form a group to participate in the experiment. Studying gender effects is beyond the scope of this study.

4. State that the sample is composed of 30 pairs

Thank you for the catch. To make it more visible, we have added this information under the “participants” section.

5. Regarding the behavioral analysis – what is the rationale behind looking at the timing of the squeezes and how they change over time? The authors communicated to the reader that the objective of this study is to look at neural bases of mediated touch. Please explain and, if appropriate, add a section in the introduction about the meaning of this.

Thanks for the comment. The squeeze analysis aims to: (1) confirm that the squeeze is properly transmitted and perceivable to the receiver, (2) analyze how the MIT communication could affect the squeeze behavior, and (3) identify potential time of interest to conduct the neural analysis.

We modified the text to clarify the objectives. We revised that point in the Behavioral analysis section.

Results:

6. "It is presumed that prefrontal alpha oscillation increases due to recognizing the 223 role of the MIT encoder and preparing for the motor task" – please provide a reference for this assumption.

We fully appreciate your comment and added the reference below:

Van der Lubbe RH, Sobierajewicz J, Jongsma ML, Verwey WB, Przekoracka-Krawczyk A. Frontal brain areas are more involved during motor imagery than during motor execution/preparation of a response sequence. *International journal of psychophysiology*. 2021;164:71–86

Discussion:

"First of all, it is worth noting that the MIT is well perceived by the communicating 286
participants. When asked whether they perceived the squeeze or not, the MIT decoder 287
confirmed that they felt the squeeze cue more than 95 % of the times (mean 98.28 %, 288
SD 2.91 %). Furthermore, during the no MIT task, both participants confirmed that no 289
squeeze is felt more than 95 % of the time (mean 95.95 %, SD 6.74 %)). This clearly 290
demonstrates that the squeeze MIT was reliably communicated and perceived between 291
the two participants".
Please move this paragraph to the results section, discussion should be about interpreting results and not reporting them for the first time there.

Thank you so much for the comment and we fully agree with your comment. Now we moved this paragraph to the behavioral evaluation in the results section.

"

differences in the squeeze behavior of the two participants at the end of the 313
experimental session may have decreased due to the increase in social coordination 314
between the two communicating parties
"

This is a very interesting finding – can the authors please elaborate why would social coordination decrease the difference in the squeeze? Please cite relevant studies to support this hypothesis.

Thank you for your useful comment. We slightly modified the text as we can't confirm this with the current study. We also added a reference to support this discussion point.

The discussion is very descriptive of the results and does not interpret them nor compares the advancement made here for past data.
This whole section should be written differently with stating the purpose of the study and the results, then discuss past studies that relate to the present study and say what this study adds to them (studies about neural synchronization of touch and neural synchronization in general), then discuss why the alpha band had the most significant results (simulation mechanisms, empathy, social cognition, social skills – it is all in the literature and there are many studies on this topic), then mention limitations and the study and then have the last paragraph.
Discuss the role of gender since you recruited both men and women for the study
Discuss the topic of familiarity since there is literature on synchrony between familiar and stranger pairs.

We would like to apologize if the discussion was not very comprehensive. Your advice is very helpful and we appreciate it. We hope you are satisfied with the revised discussion section.

**Reviewer #2**:

Wanjoo Park et al. analyzed brain synchronization between 2 participants using EEG data while one of them had to squeeze a cuff and the other one received that pressure.
The authors found differences in alpha PSD and connectivity using the PLV index. Overall the manuscript is well written, but more information on methods is needed. The lack of correction for multiple comparisons and other methodological problems make it unsuitable for publication at this time.

We thank your insightful comments. We appreciate your efforts to improve the quality of the manuscript. We have addressed your comments point by point including more information on

methods and multiple comparisons issues.

My comments are included below.

Abstract: "The findings and limitations of this study are further discussed, and perspectives on future research are provided." Instead of this statement, the main conclusion of the work should be included in the abstract.

Thanks for the comment. We added a sentence to summarize the findings of the study.

Hypothesis: No specific hypotheses are stated other than "We hypothesize that MIT increases interbrain synchrony" Therefore, it is supposed to be an exploratory work that should be accompanied by multiple comparisons correction.

Thanks for the comment. We modified the last paragraph of the introduction section to clarify our hypothesis.

Methods: Methods are written in the present tense. They are usually written in the past tense.

Your feedback is appreciated. We have reflected the changes under the Methods section.

Regarding the experimental setup, it would be interesting to indicate what is the delay from the time the pressure sensor detects changes in the sender's cuff until the other participant receives changes in pressure. In addittion, could you please indicate the pressure resolution in the decoder participant?

Since a common air channel was established between the encoder and decoder, both cuffs were inflated to a 15 psi pressure level. The transfer of pressure was almost instantaneous without any delay (less than 10 ms), however, the response time had no effect on the protocol since the pressure controller was not activated during the MIT communication (it was activated between trials). The pressure feedback system was built using one pressure sensor to control the overall pressure of the common air channel. We added these details to the experimental setup description section.

From the picture it does not look like the participants were wearing headphones to isolate them from external noise. Did the setup (especially the air pump or valves) produce a lot of noise that could affect the participants, and in turn synchronize their brain activity with such noise?

The air pump was placed in an acoustic container and away from the participants during the experiment. Also, it is important to note that the pump was only activated between the subsequent trials to minimize the interference in the recorded pressure measurements.

We have elaborated more on this under the “Experimental setup” section.

Fig 3: please indicate the duration of the auditory cues.

Thanks for the comment. The duration was 100ms, we added this information to Figure 3.

Line 100: "The application is also responsible to record and store the pressure profile of the participants during the experimental task in a data file". I understand that you have the pressure profiles of both the "encoder" and the "decoder" participant, could you show the profiles in figure 4 separating decoder and encoder? this would help to know the delay between both.

Thanks for the comment. Please note that we utilized a single pressure sensor. Figure 4 shows the reading for the pressure sensor over the squeeze event (the pressure would be the same in the air channel regardless of who’s the encoder and who’s the decoder).

Line 182: "Topography analysis for the four frequency bands was conducted". It is not clear at which scalp locations the PSDs of the different frequency bands are compared. The authors might consider performing a time-frequency analysis instead of PSDs every 200 ms. In general, it would be necessary to go deeper into how the analyses were performed.

We apologize if the wording was not very clear. Topography analysis was performed for each frequency band and the whole brain was considered. PSDs were calculated at each sampled time point and the 200 ms time interval was for the visual presentation. To clarify this, we have revised this part in the EEG analysis and functional connectivity section.

Lines 190-193: This sounds like double-dipping. The authors first look for the time window with

statistical differences and then analyze this time window a second time. I believe that this is not a correct way to perform the analysis.

We actually wanted to look for TOIs and then look for regions of the brain that showed significant changes in interbrain synchrony within those TOIs, but that could be a double-dipping. To avoid double-dipping, we looked for a TOI that showed meaningful differences across all the conditions in the whole brain as compared to the baseline. The results are shown in Figure 6. Figure 7 demonstrates the same finding in the form of a box plot. We also agree that some of the wording may be confusing. Thus, we have revised it to make it clearer.

Results: Again, the absence of correction for multiple comparisons is perhaps the most limiting factor of the manuscript. Many comparisons are performed without specific hypotheses and there is no correction for false positives.

We fully agree with your comment. We corrected the *p*-values in Figure 6 using the false discovery rate (FDR) and updated the figure/caption. Also, we moved the alpha interbrain synchrony figure to the appendix section since this is not our primary finding.

Fig 6: This figure should include the whole interval, like the other figures, up to 2500ms. How is it possible that there are differences between MIT and non-MIT before somatosensory stimulation occurs? In the discussion the authors interpret this as an index of social coordination. However, it looks to me like an index of readiness for a condition in which they must perform a task. Probably the brain activity of both participants are synchronized to the auditory cue in the active condition, which requires more attentional resources than in the non-MIT condition, in which participants do not have to do anything. Makes more senso to me that such synchronization is the result of both participants reacting to the sound of the cue, irrespective of social interaction.

Thank you for your comment. We analyzed the entire 2500 ms and decided to show the time course of alpha PLV up to 500 ms in Figure 6 to illustrate the time interval of significance better. The FDR control was used to correct for multiple comparisons over the entire 2500 ms. We modified the figure caption to clarify this.

As you say, the window of significant difference between MIT and no MIT is very short. In the MIT task, the moment the encoder recognizes the cue and moves the cuff, it is interpreted as an increase in the alpha PLV between the two participants. Since the visual and auditory cues in the

MIT decoder and no MIT tasks are identical, the decoder in the MIT task cannot distinguish between the MIT and no MIT conditions using the visual and auditory cues. This is supported by the increase in the alpha PLV index in the no MIT condition in Figure 6. We have modified Figure 3 to show that the visual and auditory cues in the no MIT task are the same as the decoder in the MIT task, so there is no confusion.

In the interpretation of Fig 7: "When the average of the PLV indices between the EEG channels of two participants during TOI increased compared to the baseline, it was expressed in red solid line, and when it decreased, it was expressed in a blue dashed line in Figure 7. It can be seen that the interbrain functional connectivity increased significantly in the case of MIT compared to the case of No MIT" The authors should be careful with this interpretation, they have not compared between conditions, only TOI vs baseline, thefore they cannot say that there were significant differences between conditions.

We apologize if we've confused you with inaccurate wording. We have modified the last sentence of the Results section to clarify this.

Line 266: t and p-value should be included.

Sure. We have added the *t* and *p* values.

Discussion: Alpha and beta are two key somatosensory frecuencies. The authors could discuss at some point why no PSD differences are observed in beta.

As you mentioned, alpha and beta frequency bands are highly correlated with motor movements and we also expected results in the beta frequency band. However, the existing literature shows that alpha and beta functional connectivities between brains depend more on the interaction between two people than on the motor movement itself [reference below]. We have added this to the discussion section as per your suggestion.

De Vico Fallani F, Nicosia V, Sinatra R, Astolfi L, Cincotti F, Mattia D, et al. Defecting or not defecting: how to “read” human behavior during cooperative games by EEG measurements. PloS one. 2010;5(12):e14187.

Line 287: It would be more appropriate to include this information in the results than in the discussion.

Thank you so much for the comment and we fully agree with your comment. Now we moved this paragraph to the behavioral evaluation in the results section.

---

6. PLOS authors have the option to publish the peer review history of their article (what does this mean?). If published, this will include your full peer review and any attached files.

If you choose "no", your identity will remain anonymous but your review may still be made public.

**Do you want your identity to be public for this peer review?** For information about this choice, including consent withdrawal, please see our Privacy Policy.

Reviewer #1: No

Reviewer #2: No

**Again, thank you very much for your kind and excellent comments.**
**We hope our responses are satisfactory enough,**
**and hope to hear positive news from you.**
